# Transceptors at the boundary of nutrient transporters and receptors: a new role for *Arabidopsis* SULTR1;2 in sulfur sensing

**DOI:** 10.3389/fpls.2014.00710

**Published:** 2014-12-11

**Authors:** Zhi-Liang Zheng, Bo Zhang, Thomas Leustek

**Affiliations:** ^1^Plant Nutrient Signaling and Fruit Quality Improvement Laboratory, Citrus Research Institute, Southwest University, Chongqing, China; ^2^Department of Biological Sciences, Lehman College, City University of New York, Bronx, NY, USA; ^3^Department of Plant Biology and Pathology, Rutgers University, New Brunswick, NJ, USA

**Keywords:** sulfate, SULTR1;2, transporter, sensor, transceptor

## Abstract

Plants have evolved a sophisticated mechanism to sense the extracellular sulfur (S) status so that sulfate transport and S assimilation/metabolism can be coordinated. Genetic, biochemical, and molecular studies in *Arabidopsis* over the past 10 years have started to shed some light on the regulatory mechanism of the S response. Key advances in transcriptional regulation (SLIM1, MYB, and miR395), involvement of hormones (auxin, cytokinin, and abscisic acid) and identification of putative sensors (OASTL and SULTR1;2) are highlighted here. Although our current view of S nutrient sensing and signaling remains fragmented, it is anticipated that through further studies a sensing and signaling network will be revealed in the near future.

## TIGHTLY REGULATED SULFATE UPTAKE IS REQUIRED FOR SULFUR ASSIMILATION AND UTILIZATION

Plants have evolved a biosynthetic pathway to assimilate sulfate (SO_4_^2–^), a primary source of the essential nutrient sulfur (S), into Cys and Met, which are then used for synthesis of proteins and various S-containing compounds including glucosinolates and glutathione (GSH; Takahashi et al., 2011). SO_4_^2–^ is taken up from the rhizosphere by roots and is subsequently translocated into shoots. Therefore, SO_4_^2–^ transport and assimilation must be tightly coordinated to meet the dynamic demand for S. SO_4_^2–^ uptake and translocation is mediated by transporters (SULTR) with specific gene products performing distinct and also overlapping functions (Gigolashvili and Kopriva, 2014). In *Arabidopsis*, two members of group 1 (SULTR1;1 and SULTR1;2) are high affinity SO_4_^2–^ transporters and mediate SO_4_^2–^ uptake into roots (Gigolashvili and Kopriva, 2014). Several members of Groups 2 and 3 are likely involved in SO_4_^2–^ translocation from roots to shoots, while Group 4 (SULTR4;1 and SULTR4;2) functions in vacuolar export of SO_4_^2–^ (Takahashi et al., 2011; Gigolashvili and Kopriva, 2014). In response to S deficiency, many of the transporter genes are transcriptionally up-regulated. The two most studied transporters, SULTR1;1 and SULTR1;2, have been shown to act redundantly in controlling SO_4_^2–^ uptake from roots, with SULTR1;2 having a major role (Takahashi et al., 2011; Gigolashvili and Kopriva, 2014). This tightly regulated transport system is critical for plant response and adaptation to the dynamically changing S nutrient environment.

## KNOWLEDGE OF S SENSING AND SIGNALING REMAINS FRAGMENTED

To understand the regulatory mechanism of S sensing, transport and signaling, significant efforts have been made and exciting progress is summarized below.

### INSIGHTS INTO TRANSCRIPTIONAL CONTROL IN S DEFICIENCY RESPONSE

Several transcriptome profiling studies reported that more than 1500 genes in *Arabidopsis* are up-or down-regulated by S deficiency (Hirai et al., 2003, 2004; Maruyama-Nakashita et al., 2003, 2006). These studies confirmed up-regulation of SULTR1;2 and other transporter genes, and led to the identification of two novel S-responsive genes, *BGLU28* and *SDI1*, which have received considerable attentions. *BGLU28* is the most strongly up-regulated gene in several of the studies and is hypothesized to act by releasing S from glucosinolate, which is potentially a major S storage compound in the vacuole (Maruyama-Nakashita et al., 2003, 2006; Dan et al., 2007). *SDI1* is annotated as a protein similar to male sterility family protein MS5 and recent evidence suggests that its expression level can be used as a biosensor of S nutrient status (Howarth et al., 2009). Interestingly, a *cis* element has been identified called SURE that is necessary for S-deficiency control including transcriptional regulation of *BLGU28* (Maruyama-Nakashita et al., 2005). Furthermore, transcriptional regulators have been identified. The *SLIM1* mutants lack the ability to up-regulate S-response gene expression including that of *SULTR1;2* (Maruyama-Nakashita et al., 2006). Although many of S-responsive genes (including *BGLU28* and *SDI1*) are under SLIM1 control, others (e.g., *APR2* and *APR3*) were not affected, strongly suggesting that although SLIM1 may be a major S-response transcription factor, additional transcriptional regulators are also involved. Consistent with this, several MYB transcription factors, in particular *MYB28* and *MYB29* which are transcriptionally repressed by S-deficiency, have been shown critical for transcriptional regulation of genes for the biosynthesis of glucosinolate which potentially serves as a critical S storage compound (Yatusevich et al., 2010). Recently, a microRNA gene (miR395) was shown to be important for regulating several target genes involved in S-deficiency response including *SULTR2;1/AST68* and *APS4* (Kawashima et al., 2009, 2011). Interestingly, miR395 was shown to be controlled by SLIM1 (Kawashima et al., 2011). Taken together, these studies provided an important foundation for understanding the transcriptional events in the nucleus.

### EMERGING UNDERSTANDING OF THE ROLE OF PROTEIN PHOSPHORYLATION, DEGRADATION, AND HORMONES IN S DEFICIENCY RESPONSE

SULTR1;2 was shown to be regulated posttranscriptionally (Yoshimoto et al., 2007). The effects of inhibitors of protein kinase and proteasome have indicated that protein phosphorylation (Maruyama-Nakashita et al., 2004a) and degradation (Pootakham et al., 2010) are involved in regulating S transport and S-starvation response in *Arabidopsis* and *Chlamydomonas*, respectively. On the other hand, the role of hormones has been increasingly recognized as a key factor in S response. Based on surveys for the impact of several hormones on the S deficiency-activated expression of beta-conglycinin (Ohkama et al., 2002),*SULTR1;2* (Maruyama-Nakashita et al., 2004b), and *BGLU28* (Dan et al., 2007), it seems that auxin, cytokinin, and abscisic acid (ABA) are involved in negatively regulating S deficiency response. Cytokinin seems to have a broader effect in S response as all of the above three S response genes could be suppressed by exogenous application of this hormone. Furthermore, genetic evidence using a cytokinin receptor mutant *cre1* demonstrated the negative regulatory role of cytokine on S uptake (Maruyama-Nakashita et al., 2004b). The negative regulatory role of ABA was first implicated by the observed suppression by S deficiency of an ABA response marker *RD29B:GUS* and down-regulation of *BGLU28* by externally applied ABA (Dan et al., 2007). A role for ABA biosynthesis in S response was recently reported (Cao et al., 2014). Compared to ABA and cytokinin, the role of auxin in S response has received more attentions. Auxin was first implicated as a regulator of S deficiency response by the observed up-regulation of auxin-inducible genes (such as *IAA28*) and *NITs* (likely involved in auxin synthesis) under S deficiency (Nikiforova et al., 2003), although S deficiency did not significantly alter auxin level (Kutz et al., 2002). However, evidence obtained from the use of *DR5:GUS*, an auxin response marker, suggests that S deficiency inhibits auxin accumulation or response (Dan et al., 2007). Such an inhibitory effect of auxin biosynthesis was confirmed recently (Zhao et al., 2014). Furthermore, by applying auxin externally, the S deficiency-activated *BGLU28* expression is down-regulated. The role of auxin response regulators such as IAA28 and ARF-2 in controlling expression of S metabolism genes has been implicated using a transgenic approach (Falkenberg et al., 2008), and a definite role of auxin was demonstrated by two genetic studies. An auxin signaling component called AXR1, which is a component of the 26S proteasome, was shown to be involved in the S deficiency response (Dan et al., 2007), in agreement with the subsequently reported role of protein degradation in *Chlamydomonas* S response (Pootakham et al., 2010). Another S response mutant is allelic to *BIG* (a calossin-like protein involved in polar auxin transport), indicating a role for auxin transport as well as auxin biosynthesis or response in S signaling (Kasajima et al., 2007).

*Most interestingly, putative S sensors or sensing components have been reported.* Cys homeostasis is tightly controlled by the Cys synthase complex which consists of Ser acetyltransferase (SAT, the enzyme producing the substrate for Cys biosynthesis) and O-acetylserine (thiol) lyase (OASTL, the enzyme producing L-Cys; Yi et al., 2010). *Arabidopsis* OASTL has three isoforms, OASTL-A1, OASTL-B, and OASTL-C, which are located in the cytosol, plastids, and mitochondria, respectively. OASTL-A1, the most abundant isoform, has been demonstrated *in vitro* to specifically interact with the STAS domain of SULTR1;2 (Shibagaki and Grossman, 2010). Interestingly, this interaction may be physiologically relevant as demonstrated in a heterologous yeast system. The interaction could enhance OASTL-A1 Cys synthesis activity at the same time it inhibits SULTR1;2 transport activity. This reciprocal activity regulation has led to the proposal that OASTL-A1 is involved in sensing of S status (Shibagaki and Grossman, 2010). OASTL-C has also been reported to act in Cys sensing (Wirtz et al., 2012). The questions remain whether these two differentially localized OASTL members sense Cys or SO_4_^2–^ located in different compartments and how they act to sense S status.

Most recent genetic and physiological evidence obtained from our groups have shown that besides its high affinity transport function, SULTR1;2 has a novel regulatory function (Zhang et al., 2014). Using *BGLU28* promoter:GUS as a mutant screening tool, two novel alleles of *SULTR1;2* were isolated that exhibit high GUS activity even under sufficient S conditions: *sel1-15* (D108N) and *sel1-16* (G208D). These two mutations lie in the predicted transmembrane (TM) helices TM11 and TM5. In contrast to all prior studies in which up-regulation of S response genes in *sel1* mutants were interpreted as the result of compromised SO_4_^2–^ uptake and consequently lower accumulation of internal SO_4_^2–^ or its metabolites (Shibagaki et al., 2002; Maruyama-Nakashita et al., 2003; El Kassis et al., 2007), we have provided two lines of convincing physiological evidence that support the hypothesis that up-regulation of *BGLU28* and three other genes (*SULTR4;2*, *SDI1*, and *LSU1*) could be independent of the compromised SO_4_^2–^ uptake and internal S status of the mutants (Zhang et al., 2014). First, under high concentration of SO_4_^2–^ (10 mM) which did not lead to a difference in internal SO_4_^2–^ concentration and GSH level, *sel1-15/16* and a null allele (*sel1-18*) still had higher gene expression level than their wild-type (WT) backgrounds. Second, treatments with 1 mM Cys or 1 mM GSH in the SO_4_^2–^ deficiency medium (which did not lead to any difference in Cys uptake and/or internal GSH contents between the *sel1* alleles and WT) also led to higher gene expression level in *sel1-15/16/18*. These results strongly suggest that the *sel1* seedlings (in particular the expression in roots) grown under sufficient S behave as if they have been treated by certain degrees of S deficiency. In other words, the mutations in *SULTR1;2* reduce sensitivity to the S-induced suppression of S response genes. The evidence points toward a novel function for SULTR1;2 in regulating S nutrient response besides its transport function. The possibility that SULTR1;2 acts as an S sensor is discussed in the next section.

## CAN SULTR1;2 ACT AS A PUTATIVE PM-LOCALIZED SULFATE TRANSPORTING RECEPTOR?

Dual function transporters, like SULTR1;2 described above, are not unusual. Studies in yeast and animal nutrient transport and sensing have revealed the existence of classic receptors (which are not involved in transport, e.g., G-protein-coupled receptor Gpr1), transceptors (which are either transporting receptors, e.g., Gap1, or non-transporting receptors, e.g., Snf3) and the majority of common transporters (which do not have a sensing function; Thevelein and Voordeckers, 2009). Therefore, transceptors can be considered at the boundary between receptors and transporters. In general, to demonstrate a receptor function for a transporter molecule, genetic or pharmacological evidence is required that shows decoupling of nutrient transport and signaling, i.e., the signaling output is independent of transport.

**FIGURE 1:**
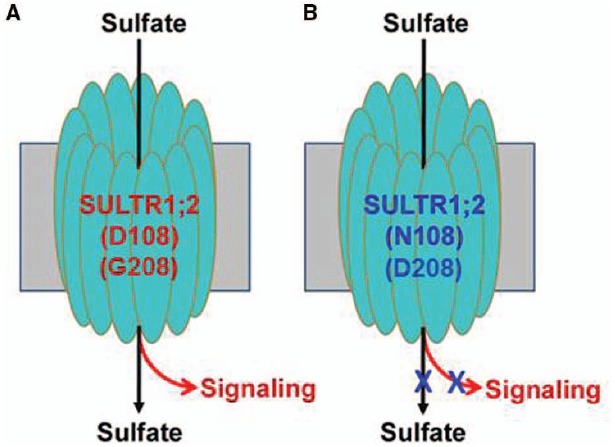
**A hypothetical model for the dual function transceptor SULTR1;2.** **(A)** The normal (wild-type) transceptor functions both in SO_4_^2–^ transport and signaling; **(B)** the transceptor is defective both in transport and signaling due to the mutations of D108N or G208D.

**FIGURE 2:**
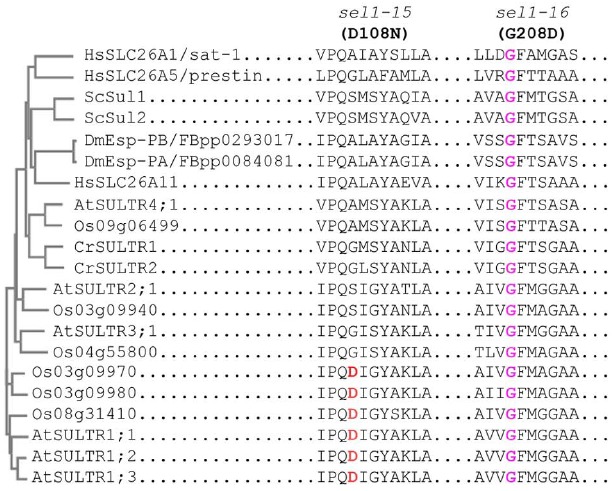
**Phylogenetic relationships of SULTR1;2 and its closely related members of transporters in representative eukaryotes.** The phylogenetic tree for SULTR1;2 and its closely related transporters, which is constructed using their full-length amino acid sequences, is shown on the left. The amino acid sequence alignment of the motifs surrounding D108 (*sel1-15*) and G208 (*sel1-16*) of SULTR1;2 and similar regions for other closely related transporters is shown on the right. At, *Arabidopsis thaliana*; Cr, *Chlamydomonas reinhardtti*; Dm, *Drosophila melanogaster*; Hs, *Homo sapiens*; Os, *Oryza sativa*; Sc, *Saccharomyces cerevisiae*.

In the case of SULTR1;2, the mutations in TM1 (*sel1-15*) or TM5 (*sel1-16*) could abolish both SO_4_^2–^ transport and signaling (as measured by expression of S response genes), but the defect in signaling could be independent of SO_4_^2–^ transport and accumulation (Zhang et al., 2014). Because of this, we propose that SULTR1;2 can function as a putative SO_4_^2–^ transceptor (Figure 1). Although SULTR1;2 cannot be the only S-sensor since the *sel1-15/16* mutants show reduced sensitivity to S but does not entirely abolish the S-limitation response, this finding provides a first intriguing insight into S-sensing in plants given its PM location where extracellular SO_4_^2–^ is first in contact with the PM-localized sensors. Note that a dual-affinity nitrate transporter called NRT1.1 has been demonstrated to act as a nitrate sensor (Ho et al., 2009; Bouguyon et al., 2012), and thus using nutrient transporters to sense the external nutrient status may be evolutionally conserved and advantageous to plants. Indeed, a phosphate transceptor (Pho84) has been reported in yeast (Popova et al., 2010). More encouraging is that in yeast SO_4_^2–^ transporters Sul1/2 have also been described as being transceptors (Conrad et al., 2014). To gain further insights into the evolutionarily conserved mechanism of using sulfate transporters as sensors, we performed a sequence alignment using transporters from *Arabidopsis*, rice, *Chlamydomonas*, yeast, *Drosophila* and humans that are most closely related to *Arabidopsis* SULR1;2. The result (Figure 2) shows that while D108 is only specific to SULTR1 group in *Arabidopsis* and rice, G208 is highly conserved in all transporters. It will be interesting to determine whether G208 is critical for SO_4_^2–^ transport and signaling in many eukaryotes.

It remains unclear how plants use SULTR1;2 to sense external SO_4_^2–^ status and adopt a high or low affinity transport system in response to dynamic S environment. However, studies from the yeast amino acid transceptor Gap1 or *Arabidopsis* nitrate transceptor NRT1.1 may provide some hints for the SULTR1;2-mediated sensing mechanism. In NRT1.1-mediated nitrate sensing and signaling, auxin transport and NRT1.1 phosphorylation have been shown to be critical (Ho et al., 2009; Bouguyon et al., 2012). In yeast, Gap1 uses the same sites for amino acid binding/transport and signaling (Van Zeebroeck et al., 2009; Conrad et al., 2014). Once amino acid is bound to Gap1, it triggers a conformational change in Gap1 that subsequently allows the amino acid be transported into the cytoplasm and in the same time a signaling cascade is activated. If the amino acid status is perceived to be sufficient, Gap1 undergoes a rapid endocytic process that removes it from the PM and sorts it for degradation.

## FUTURE PROSPECT

Exciting findings in the past 10 years have led to the identification of several components from the PM to the cytoplasm and to the nucleus that are involved in S sensing, transport and downstream response. Several outstanding questions remained to be answered. What is the SULTR1;2 topology and does SULTR1;2 have separate sensing and transport domains? Can SULTR1;2 interact with OASTL *in vivo* (if so, which OASTL isoform?) and exert the effect of S sensing *in planta*? How does SULTR1;2 link to various signaling intermediates acting at the PM, the cytoplasm or the nucleus? Are there additional partners that may form a larger SULTR1;2-based S sensing complex? If such complex cannot account for all S responses, what other sensors are involved? Further, what are the roles of these sensing components in local and systemic S signaling (Hubberten et al., 2012)? Although our current view of S sensing and signaling remains fragmented, further studies into these questions will allow us to piece together individual components and ultimately construct the SULTR1;2-mediated S sensing and signaling pathway or network.

### Conflict of Interest Statement

The authors declare that the research was conducted in the absence of any commercial or financial relationships that could be construed as a potential conflict of interest.
